# End-to-End Platform for Electrocardiogram Analysis and Model Fine-Tuning: Development and Validation Study

**DOI:** 10.2196/81116

**Published:** 2026-01-30

**Authors:** Lucas Bickmann, Lucas Plagwitz, Antonius Büscher, Lars Eckardt, Julian Varghese

**Affiliations:** 1 Institute of Medical Informatics, University of Münster, Münster, Germany; 2 Institute of Medical Data Science, Otto-von-Guericke University Magdeburg, Leipziger Str. 44, Building 2, Magdeburg, 39120, Germany, 49 391 67 13544; 3 Clinic for Cardiology II: Electrophysiology, University Hospital Münster, Münster, Germany

**Keywords:** health informatics, electrocardiogram, machine Learning, deep learning, end-to-end platform

## Abstract

**Background:**

Electrocardiogram (ECG) data constitutes one of the most widely available biosignal data in clinical and research settings, providing critical insights into cardiovascular diseases as well as broader health conditions. Advancements in deep learning demonstrate high performance in diverse ECG classification tasks, ranging from arrhythmia detection to risk prediction for various diseases. However, the widespread adoption of deep learning for ECG analysis faces significant barriers, including the heterogeneity of file formats, restricted access to pretrained model weights, and complex technical workflows for out-of-domain users.

**Objective:**

This study aims to address major bottlenecks in ECG-based deep learning by introducing ExChanGeAI, an open-source, web-based platform designed to offer an integrated, user-friendly platform for ECG data analysis. Our objective is to streamline the entire workflow—from initial data ingestion (regardless of device or format) and intuitive visualization to privacy-preserving model training and task-specific fine-tuning—making advanced ECG deep learning accessible for both clinical researchers and practitioners without machine learning (ML) expertise.

**Methods:**

ExChanGeAI incorporates robust preprocessing modules for various ECG file types, a set of interactive visualization tools for exploratory data analysis, and multiple state-of-the-art deep learning architectures for ECGs. Users can choose to train models from scratch or fine-tune pretrained models using their own datasets, while all computations are performed locally to ensure data privacy. The platform is adaptable for deployment on personal computers as well as scalable to high-performance computing infrastructures. We demonstrate the platform’s performance on several clinically relevant classification tasks across 3 external and heterogeneous validation datasets, including a newly curated test set from routine care, evaluating both model generalizability and resource efficiency.

**Results:**

Our experiments show that de novo training with user-provided, task-specific data can outperform a leading foundation model, while requiring substantially fewer parameters and computational resources. The platform enables users to empirically determine the most suitable model for their specific tasks, based on systematic validations, while lowering technical barriers for out-of-domain experts and promoting open research.

**Conclusions:**

ExChanGeAI provides a comprehensive, privacy-aware platform that democratizes access to ECG analysis and model training. By simplifying complex workflows, ExChanGeAI empowers out-of-domain researchers to use state-of-the-art ML on diverse datasets, democratizing the access to ML in the field of ECG data. The platform is available as open-source code under the Massachusetts Institute of Technology (MIT) license.

## Introduction

### Background

Deep learning methods applied to Electrocardiogram (ECG) analyses have demonstrated their potential as practice-changing diagnostic tools, providing critical insights into heart-related diseases [1-3]. While the push for newer technologies and improved performance metrics is essential, ensuring these advancements are accessible for general use is equally important. Tools like ChatGPT (OpenAI) have demonstrated the potential for a broad and easy application of artificial intelligence (AI), allowing users to leverage sophisticated technologies without in-depth expertise. Clinician-researchers who seek to apply machine learning (ML) to assess its potential benefits should have access to solutions that facilitate exploration and application without requiring extensive technical knowledge from data handling to data analysis. To address these challenges, there is a need for a comprehensive, end-to-end platform that integrates currently fragmented technical steps like ECG-specific data handling, preprocessing, data visualization, and model training, including transfer learning that requires cumbersome manual scripting. This would enable a seamless workflow from data loading to model deployment and would not only empower researchers to apply and train or fine-tune deep learning models for ECG analysis without programming but also facilitate reproducibility. Moreover, existing pretrained models for ECG data may not disclose model weights, which presents significant challenges (refer to “Related Work” below). While open weights empower users to use and adapt the model, they also promote reproducibility and transparency [4,5].

Additionally, the broad use of medical data is crucial for the advancement of personalized and specialized medicine but inhibits some immediate risks, such as data breaches [6]. As datasets continue to grow and come from diverse sources, ensuring data security becomes increasingly complex. To address this challenge, specialized decentralized learning techniques, such as federated learning or swarm learning, allow valuable insights to be gained without directly sharing sensitive data [7]. Furthermore, establishing uniform data standards can simplify data handling, reduce technical barriers related to varying data formats, and eliminate the need for programming, thereby making advanced analytics accessible to a broader range of users.

In this work, we introduce a novel open-source end-to-end platform for 12-lead ECGs called ExChanGeAI that streamlines essential steps of ECG analysis: (1) data loading and preprocessing of multiple input formats, (2) manual and computer-aided analysis of ECG waveform data, (3) one-click fine-tuning of classification models, allowing users to train and customize ML models with no prior expertise, (4) the trained models use the cross-platform industry-standard Open Neural Network Exchange (ONNX), enabling deployment in every instance of ExChanGeAI and facilitating the exchange of custom models across different instances, and (5) prediction of diseases with and without using pretrained models. Model sharing is supported via an integrated and adaptable WebDav file server called Model ExChanGe. The platform is built upon the principle of open-source code and open*-*weights, offering full transparency and control, empowering users to contribute to the advancement of ECG analysis models.

### Related Work

Multiple studies and reviews have addressed ECG classification and have shown that fine-tuning and transfer learning improve classification results [8-10]. A study has reported improved model accuracy by fine-tuning networks trained on diverse datasets, demonstrating enhanced performance transitioning to smaller datasets [11]. However, the used data and pretrained models were not shared. Another study used transfer learning with convolutional neural networks (CNNs) for atrial fibrillation classification, pretraining on large public datasets and fine-tuning on smaller sets, achieving performance gains [9]. While code was available, pretrained models were not shared, and usability remains a significant barrier. Multiple reviews have summarized ECG analysis pipelines and deep learning methods, such as detailed essential pipeline steps [12] and reviews of techniques like CNNs and recurrent neural networks for arrhythmia classification [13]. The SelfONN model [14] showed competitive performance in general ECG classification on PTB-XL (Physikalisch-Technischen Bundesanstalt-extra large [National Metrology Institute of Germany]) but lacked resource sharing. Various types of autoencoders, including low-rank attention [15], long short-term memory [16], adversarial [17], and denoising [18] approaches, have been explored for feature extraction, anomaly detection, and noise handling. The low-rank attention autoencoder reported high accuracy on 2 datasets by focusing on spatial features. ECG-NET, based on long short-term memory, proclaimed high accuracy for arrhythmia classification on a single database in beat-based validation. An adversarial autoencoder with a temporal CNN published superior scores of anomaly detection for 2 datasets. The attention-based denoising autoencoder improved noisy ECG signal reconstruction. However, limitations across these studies include dataset dependence, restricted generalizability, lack of publicly available pretrained models and code, and validation variability.

In a recent study leveraging the gold-standard PTB-XL [19,20] dataset, the performance characteristics of multiple deep learning models were evaluated across a spectrum of training-data sizes [21]. Notable findings indicated that the InceptionTime and XceptionTime architectures [22,23] exhibited particularly compelling performances. Specifically, InceptionTime demonstrated superior efficacy when trained with smaller datasets, whereas XceptionTime surpassed all other models in performance as training dataset size increased. This suggests a potential trade-off between model complexity and data requirements for optimal diagnostic accuracy in this domain. Due to the demonstrated strength in low- and high-data scenarios, these leading architectures for ECG analysis are highly relevant for evaluation and inclusion in the platform, particularly in contexts where training data availability may vary, such as in medical contexts.

There have also been several claimed foundation models in the field of ECG classification [24]. To the best of our knowledge, these, however, are trained on a singular database [25] and are yet undisclosed or have closed-source code and weights [26,27] in general. In one case, the published weights are different from the original model of the paper due to privacy concerns [28]. A request to publish another trained model has been declined due to intellectual property and legal concerns [29]. They are trained with techniques, such as contrastive and masked learning. This allows for unsupervised training, but restricts the learning to the latent space. For downstream tasks, such as classification, fine-tuning is required. The publications report high scores for classification; however, additional tasks are not available in the published model. While these models mark significant progress in the field, they often grapple with issues, such as overfitting to specific datasets, limited scalability, or insufficient handling of the variability and quality complications intrinsic to diverse ECG datasets.

Despite advances in ECG analysis and deep learning, the current workflows remain complex, requiring manual data transformation, preprocessing, and the use of separate tools for the visualization and analysis of ECGs, as well as for training and fine-tuning deep learning models. This fragmentation multiplies technical burdens, hinders reproducibility, and acts as a major barrier for widespread clinical adoption. Some frameworks exist to reduce the boilerplate of ML, such as the graphical tool Orange (University of Ljubljana) [30]. It does not require code, yet the workflow has to be set up manually per drag-and-drop, and it does not include any of the ECG analysis tools, such as QRS detection. In the case of AutoML tools, such as GAMA (General Automated Machine learning Assistant; originally developed by Pieter Gijsbers and Joaquin Vanschoren at the Eindhoven University of Technology) [31], it still remains code-centric. Data ingestion, for both methods, is not available out-of-the-box, such as for Digital Imaging and Communication in Medicine (DICOM), even ignoring the sampling rate, lead order, and other inconsistencies across datasets. Both tools remain unsuitable for end-to-end ECG analysis and training of ML models without substantial ML or data scientist expertise. Our work directly addresses this gap with ExChanGeAI, an integrated, accessible, and containerized end-to-end platform. This platform facilitates the visualization, transformation, prediction, and fine-tuning of deep learning models specifically for ECG data. It can leverage pretrained models, and it supports a broad range of formats and preprocessing steps, ensuring usability across different clinical and research settings. ExChanGeAI serves as a valuable resource for researchers, enabling efficient training and fine-tuning of deep learning models while preserving data privacy. This enhances both the accessibility and utility of advanced ECG analysis.

## Methods

### Overview

This section introduces the ExChanGeAI platform, a fully containerized, interoperable, and standardized end-to-end platform for ECG analysis, diagnosis prediction, and model fine-tuning. It is designed for nonexpert users, enabling advanced AI-enabled workflows in a unified interface without requiring specialized technical expertise. The open-source code is freely available under the MIT license [32].

### ExChanGeAI Platform

**Figure 1.**
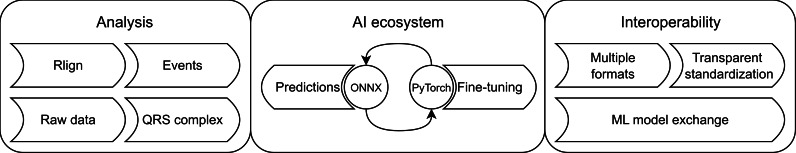
Overview of the capabilities of the end-to-end platform ExChanGeAI and its three main distinct parts: (1) Analysis, (2) The Artificial Intelligence Ecosystem, and (3) Interoperability. AI: artificial intelligence; ML: machine learning; ONNX: Open Neural Network Exchange.

ExChanGeAI is a containerized web application providing an integrated suite of AI-enabled ECG analysis tools for researchers and clinicians. The platform merges human expert analysis capabilities and AI predictions in a unified, interactive end-to-end platform (refer to Figure 1). The functionalities include (1) signal analysis, (2) model-based prediction, (3) model exchange and repository, and (4) semiautomated training and fine-tuning.

The platform is open-source, using the standardized ONNX model format and additionally supports and encourages the open-weights practice of ML models [4]. The platform consists of multiple views with different foci. The analysis view integrates the visualization of waveforms of raw signals, QRS complexes, and events (fiducial points), computed transparently by Neurokit2 [33]. Additionally, precise R-peak alignment and median beat transformations are supported through the integration of the recently published ECG-preprocessing package, Rlign [34]. These data transformations can be exported in different formats for further research. The platform focuses on resting 12-lead ECGs, displayed in a 2x6 grid in mV scale, and integrates general spatial transformations, including zooming with synchronized adaptation across all leads. This signal view has been designed in collaboration with cardiologists for their everyday use. For visualizing QRS complexes and events, lead II is conventionally applied. The prediction view uses selected models to predict diagnoses and other targets—such as QTc—based on raw signal data, offering a table with predicted diagnosis probabilities (or arbitrary keys), highlighted with a clear color-coding scheme. Dataset distributions, confusion matrices, and class-wise receiver operating characteristic curves can be computed on the platform itself, including suggested thresholds for class-specific Fmax scores. This enables researchers to optimize the threshold for each specific dataset and the health care providers’ requested strategy. These differ gradually and can focus on either specificity or sensitivity, depending on the classification targets. Models are provided within the platform using an integrated and interchangeable file server, which enables the crucial aspect of model sharing (see section “Interoperability and Model Sharing”). Currently, we provide multiple models for four targets: (1) diagnostic superclasses, (2) anterior and inferior myocardial infarction (MI), (3) diverse bundle branch blocks, and (4) revascularization need (refer to section “Results”). Researchers can also train specialist models based on their own labeled data. The training and fine-tuning require no prior knowledge of ML. Currently, only 12-lead ECG data with corresponding labels are required. The backend is engineered for high performance and scalability using asynchronous views, multithreading, and full compute unified device architecture GPU (graphics processing unit) support, including multi-GPU data-parallel training for large-scale fine-tuning.

### Data Loading and Preprocessing

**Figure 2.**
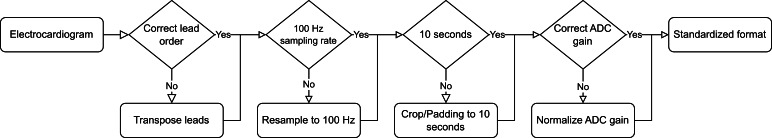
Flowchart of the preprocessing applied to any electrocardiogram data while being loaded into the application, independent of the file format. ADC: analog-to-digital converter.

ExChanGeAI is designed to handle the variability of real-world ECG data. It supports various ECG input formats, across all possible sampling rates, CSV files (.csv), NumPy arrays (.npy, .npz), DICOM-stored WaveformSequences (.dcm), MATLAB (MathWorks) formatted data (.mat), general DAT files (.dat), and XML files (.xml). Major research and clinical ECG standards (PhysioNet - DAT, UK Biobank - XML) are supported. For ExChanGeAI, all data are normalized by resampling frequency signals to a configurable unified frequency target (defaulting to 100 Hz) using the Fast Fourier Transform, as previous work has shown that the sampling rate does not notably decrease the performance of ML models, but reduces computational overhead manifold [35-38]. This parameter is fully configurable, allowing users to adjust the sampling rate in a local deployment to match specific requirements. Signals not conforming to the standardized format (12-lead, 10-second waveform) are adjusted via expansion or cropping, and all scales are automatically standardized to millivolt (mV) with a 1000 analog-to-digital converter units gain, if necessary (refer to Figure 2).

### Interoperability and Model Sharing

The platform enables training of new models in a secure and privacy-preserving manner. Still, as seen with many publications in the medical domain, pretrained models are not made public [26,29] or depend on external libraries and require specific versions [28]. To promote the open and interoperable ML standard, this work adopts the ONNX as the primary used format. Therefore, our platform is compatible with all ONNX models, honoring the current operation set (Opset 20 and below), and PyTorch models, if the given model structure is provided alongside. The models are not specified with any special requirements, except for a dynamic batch size export. With the use of ONNX, this work aims to ensure that the trained model is widely accessible and interoperable. Therefore, a model sharing interface, called Model ExChanGe*,* is integrated into the platform, where curated pretrained models are automatically synced and made available for prediction as well as fine-tuning. Additional models can be published into the repository, or your own WebDav instance can be set up.

By default, ExChanGeAI provides 3 baseline model architectures—each as pretrained and untrained models. This includes the XceptionTime [23], InceptionTime [22], and the PhysioNet/CinC Challenge 2021 12-lead second-best model, DSAIL SNU (Data Science & Artificial Intelligence Laboratory Seoul National University [19,39,40])—the best model did not provide weights. Additional models can be incorporated by uploading them into the platform or using the default model exchange file server. We incorporate all evaluated models to extend the research community with open-source and pretrained model weights.

The adoption of the ONNX industry-standard model format ensures that ExChanGeAI is not limited to a proprietary ecosystem. Models trained or fine-tuned with ExChanGeAI can be seamlessly imported, shared, or deployed across different institutions and environments, whether in research settings or clinical contexts. This plug-and-play capability allows users to leverage pretrained models, contribute their own, or integrate compatible architectures developed externally, with zero code changes and minimal configuration. As a result, the platform not only accelerates collaboration but also supports sustainable, evolving workflows as new models and data become available.

### Fine-Tuning Platform

**Figure 3.**
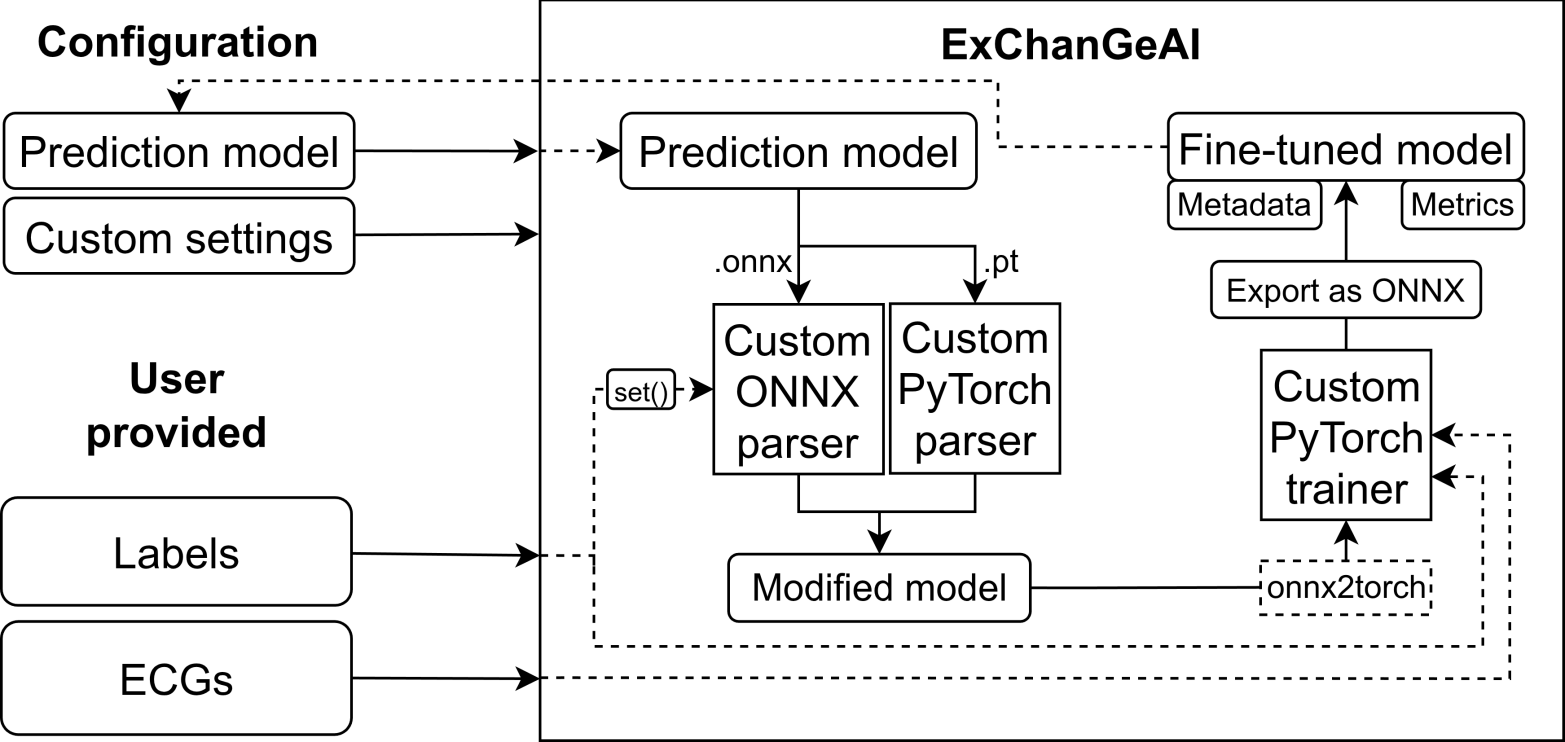
Overview of the semiautomatic fine-tuning process in the backend of ExChanGeAI. ECG: electrocardiogram; ONNX: Open Neural Network Exchange.

ExChanGeAI provides an interactive user interface for fine-tuning with user-supplied data, abstracting all low-level ML steps, and facilitating the exchange of prediction models among researchers. To ensure broad compatibility with various models and platforms, we natively support PyTorch and developed a custom parser for ONNX models to adapt the computation graph where applicable. ExChanGeAI is built upon PyTorch [41], which facilitates on-device training and leverages the ONNX framework during inference. ONNX’s custom training runtime does not support all PyTorch operators with corresponding gradient implementations, such as functions like ReduceMin and diverse Pooling Operators (Opset≤18). While defining custom operators may solve this, it requires detailed implementation knowledge for each unknown operator, creating a significant barrier for practical applications. To overcome these limitations, we use onnx2torch to convert our uniquely parsed ONNX models into PyTorch models. This conversion enhances compatibility, allowing training across different ONNX Opset versions and using the extensive feature set of PyTorch. Crucially, our parser automatically adapts the classification head to the new number of classification targets. These conversion steps are computed independently for each fine-tuning process and are entirely transparent to the user, without requiring any programming knowledge (refer to Figure 3). We provide two distinct training methods, including fine-tuning the classification heads, where the majority of the model weights are frozen, or training the entire model. Both methods use pretrained weights if a pretrained model is selected. The freezing of weights is handled automatically in the background, only allowing modification of the unfrozen weights or those belonging to the classification head.

The training and fine-tuning process uses by default the AdamW optimizer [42], due to its better generalizability and convergence than the default Adam optimizer [43], and the ExponentialLR learning rate scheduler (*γ*=0.9). Adaptive optimization algorithms tend to be more robust, have faster convergence, and therefore improve resource usage [44]. Other optimizers, such as variants of Adam and stochastic gradient descent, are available as alternatives. Initially, a learning rate finder is executed to automatically determine the optimal initial learning rate based on the provided model and data. It has been shown that this method improves the convergence speed and reliability [45]. Furthermore, the loaded data is automatically split into stratified training/evaluation sets with an 80/20% distribution. We limit the training process to a default maximum of 50 epochs and incorporate checkpointing for models with the lowest weighted validation loss, alongside early stopping. Advanced settings, such as other optimizers, batch size, number of epochs, maximum initial learning rate, and gamma can be adapted via the user interface, if required. The best model, in addition to training and evaluation statistics, is exported and downloaded after completion. To ensure comprehensive reporting and documentation, the exported statements include (1) the number of samples, (2) distribution and corresponding labels, (3) the used base model, (4) the training and evaluation loss per epoch, and (5) the corresponding *F*_1_-scores on the evaluation set.

### Evaluation

To thoroughly assess the reliability and performance of ExChanGeAI's training process, we conduct a series of scenario-based tests. The training and prediction capabilities are one of the key features of this work and are therefore mainly evaluated via training and fine-tuning of classification models on various tasks and tests on internal and external datasets using the ExChanGeAI platform, where possible. This benchmarking is crucial for validating the platform’s core value proposition (1) to empower users, even without ML expertise, and (2) to rapidly develop and deploy accurate prediction models for ECG analysis. Demonstrating robust performance across diverse datasets reinforces the platform’s usability and reliability, ultimately building trust and accelerating adoption among clinicians and researchers.

### Datasets

To train and fine-tune deep learning models, we use multiple targets and use the large open-access gold standard PTB-XL dataset [20]. In order to demonstrate model training under data-scarce conditions, the provided stratified fold 9 is used for training and fold 10 for intradataset testing. Age and sex, while technically predictable from ECG signals, are generally of limited clinical relevance in the context of model development, as these attributes are readily and reliably obtained through direct patient observation rather than requiring inferential prediction from ECG data.

The comparison includes a wide-ranging spectrum of ischemic heart diseases, structural heart diseases, and conduction abnormalities: (1) broad diagnostic categories, including MI, ST/T changes, conduction disturbances, and hypertrophy, (2) specific comparisons, such as anterior MI vs inferior MI, and diverse types of bundle branch blocks, including complete left (CLBBB), complete right (CRBBB), and incomplete left (ILBBB). The label distribution of PTB-XL is outlined in the Multimedia Appendix 1. A comparison baseline XceptionTime model is trained on the folds 1‐8 of PTB-XL for all targets to outline the training performance under data-rich conditions. We also compare our baseline model against the benchmark scores of InceptionTime from the PTB-XL benchmark paper [46]. To facilitate a direct comparison of the platform’s one-click training capabilities with the benchmark paper, we additionally trained an InceptionTime model on folds 1‐8 of PTB-XL, focusing on the superclasses.

To analyze the applicability of models across sites, we evaluate all models on interdatasets based on Yang et al [47,48], MIMIC-IV-ECG (Medical Information Mart for Intensive Care IV Electrocardiogram) [49], and Emergency Department Münster (EDMS) [38], which is an entirely new dataset from our hospital site. This helps to gauge the model’s generalizability across different ECG recordings. The latter two datasets demonstrate a relatively balanced distribution with respect to age and sex, whereas the former dataset is predominantly male and represents the smallest sample size among the 3 test sets (Multimedia Appendix 2). We selected these datasets to ensure both clinical relevance and diversity in our evaluation. EDMS is a newly collected internal dataset derived from routine clinical care, providing contemporary, real-world ECG data. MIMIC-IV ECG represents one of the largest publicly available routine care datasets, enabling robust large-scale analyses. PTB-XL serves as the gold standard for annotated ECG data, offering high-quality expert labels, while the Yang et al dataset provides a similarly gold-standard resource from an entirely different geographical region, allowing us to assess model generalizability across populations. To prevent patient leakage, only one record per patient was kept in PTB-XL before the stratified split.

Some classes present slight variations, such as MIMIC and EDMS, which do not have descriptive ECG statements but general *ICD-10* (*International Classification of Diseases, 10th Revision*) codes, which are not necessarily based solely on the given ECG. We extract the signals with the corresponding fitting maps and merged superclasses. The corresponding *ICD-10* codes, or included statements, are given for each map (refer to Table 1). This includes changes such as that bundle branch blocks (BBBs) are only encoded and divided into left- and right-BBB. We evaluate the prediction performance accordingly, counting complete- and incomplete-RBBB as the superclass RBBB. Physionet classes, from the pretrained models, are mapped to the BBB and superclasses where applicable, as no MI classes are included. For MIMIC and EDMS, in instances of multiple ECGs of the same patient, only the first record was used to negate any multiple patient testing.

**Table 1.** Matched superclass and bundle branch block statements of PhysioNet 2021 and Emergency Department Münster to PTB-XL (Physikalisch-Technischen Bundesanstalt-extra large [National Metrology Institute of Germany]) classes.

Classes	Superclasses		Bundle branch blocks		
PTB-XL^a^	CD^b^	STTC^c^	CLBBB^d^	CRBBB^e^	IRBBB^f^
PhysioNet 2021	BBB^g^, CLBBB|LBBB^h^, CRBBB|RBBB^i^, IRBBB, IAVB^j^, LAnFB^k^, NSIVCB^l^	TAb^m^, TInv^n^, LQT^o^	CLBBB|LBBB	CRBBB|RBBB	IRBBB
EDMS^p^	—^q^		LBBB	RBBB	

a Physikalisch-Technischen Bundesanstalt-extra large.

b CD: conduction disturbance.

c STTC: ST/T Changes.

d CLBBB: complete left bundle branch block.

e CRBBB: complete right bundle branch block.

f IRBBB: incomplete right bundle branch block.

g BBB: bundle branch block.

h LBBB: left bundle branch block.

i RBBB: right bundle branch block.

j IAVB: first-degree atrioventricular block.

k LAnFB: left anterior fascicular block.

l NSIVCB: nonspecific intraventricular conduction disorder.

m Tab: T wave abnormal.

n TInv: T wave inversion.

o LQT: prolonged QT interval.

p EDMS: Emergency Department Münster.

q Not available.

Table 2 shows a comprehensive overview of all extracted targets, the number of samples, and mapped *ICD-10* codes across the different external test datasets. Most datasets are uncurated and reflect real-world implications, in contrast to semicurated datasets, such as PTB-XL. There may be bad quality data, as well as discrepancies between *ICD-10* codes and mapped classes. The codes may be based on other electronic health data than the ECG, and differences may occur due to indifference between suspected and confirmed diagnoses. Label noise, which is only present in MIMIC and EDMS, may lead to underestimation of classification performance [50]. This, however, allows us to compare the models across real-world data, showing possible impact on clinical care. An additional case study with a manually annotated gold standard is evaluated on the internal EDMS dataset. To demonstrate the advanced classification task of revascularization (“does the patient require revascularization?”), which is not available in PTB-XL, the models are trained and fine-tuned using the new EDMS dataset. The labels of revascularization are case-based if the patient has been treated with a revascularization. It consists of 240 positive and negative cases each, whereas negative cases are only a stratified subset of the complete annotated dataset. An additional stratified subset (20%) of these data points is kept as testing data.

**Table 2.** External test datasets and their class distribution across all categories, their mapping from diagnostic statements or *ICD-10* (*International Classification of Diseases, 10th Revision*) codes to PTB-XL (Physikalisch-Technischen Bundesanstalt-extra large [National Metrology Institute of Germany] classes, and the data format, including sampling rate, analog-to-digital converter gain, and additional annotations.

Publication, notes, and target	Classes
Yang and Feng [48]	
500 Hz based on dataset labels	
Superclasses	HYP^a^ (HEH^b^): 647 CD^c^ (IAVB^d^, IIAVB1^e^, IIIAVB^f^, BBB^g^, LAFB^h^, NICD):^i^ 1974
Bundle branch blocks	CLBBB:^j^ 43 CRBBB:^k^ 328 IRBBB:^l^ 1051
MIMIC-IV-ECG^m^	
500 Hz with 200 adu/mV gain based on *ICD-10^n^* codes	
Superclasses	HYP (I11, I51.7): 500 MI^o^ (I21, I22): 500 CD (I44): 500
Myocardial infarcts	AMI^p^ (I21.0): 500 IMI^q^ (I21.1): 500
Bundle branch blocks (variation)	LBBB^r^ (I44.7): 500 RBBB^s^ (I45.1): 500
EDMS^t^	
100 Hz based on ICD-10^n^ codes	
Superclasses	HYP (I11, I51.7): 149 MI (I21, I22): 302 CD (I44): 255
Myocardial infarcts	AMI (I21.0): 51 IMI (I21.1): 43
Bundle branch blocks (variation)	LBBB (I44.7): 73 RBBB (I45.1): 48
500 Hz annotated through cardiologists	
Revascularization (20% test subset)	Yes: 48 No: 48

a HYP: hypertrophy.

b HEH: heart enlargement and hypertrophy.

c CD: conduction disturbance.

d IAVB: first-degree atrioventricular block.

e IIAVB: second-degree atrioventricular block.

f IIIAVB: third-degree atrioventricular block.

g BBB: bundle branch block.

h LAFB: left anterior fascicular block.

i NICD: nonspecific intraventricular conduction disturbance.

j CLBBB: complete left bundle branch block.

k CRBBB: complete right bundle branch block.

l IRBBB: incomplete right bundle branch block.

m MIMIC-IV-ECG: Medical Information Mart for Intensive Care IV Electrocardiogram.

n *ICD-10: International Classification of Diseases, 10th Revision.*

o MI: myocardial infarction.

p AMI: anterior myocardial infarction.

q IMI: inferior myocardial infarction.

r LBBB: left bundle branch block.

s RBBB: right bundle branch block.

t EDMS: Emergency Department Münster.

### Model Selection

We use the best-performing models based on the aforementioned previous research [21]. The study has shown that the InceptionTime performs well with less data in comparison to XceptionTime, but its performance lags behind when larger datasets are used. Therefore, we train a baseline model on XceptionTime, as its capability exceeds InceptionTime due to the large amount of data available for the baseline model. In comparison to the InceptionTime and XceptionTime models, we evaluate the DSAIL SNU PhysioNet 2021 model [39,40,51], the PhysioNet 2021 competition leader with available weights, and the only available foundation model, ECG-FM (Electrocardiogram Foundation Model) [28]. We aim to assess the effectiveness and improvements gained using ExChanGeAI’s training and fine-tuning capabilities and the possible use of pretrained models, especially in resource-constrained environments with very few data points.

### Preprocessing and Training

We evaluated the various architectures using two training strategies: (1) fine-tuning only the classification head and (2) training all layers. Trainings were conducted using the default settings of ExChanGeAI (commit number 4d862c04) to maintain consistency and integrity.

Xception and InceptionTime models are trained de novo (from random initialization) using non-normalized ECG data, which has been internally validated to achieve higher performance. ECG-FM and DSAIL SNU are pretrained models on PhysioNet 2021 labels, which were then fine-tuned for each classification target on PTB-XL fold 9 to demonstrate fine-tuning capabilities. In a special case, to showcase the capability of cross-task transfer learning, a pretrained XceptionTime model (superclasses with PTB-XL folds 1‐8) was separately fine-tuned on the revascularization task.

The ECG-FM foundation model’s training data details are unknown, though it is based on PhysioNet 2021 (which includes PTB-XL), limiting the validity of intradataset evaluation. The “physionet_finetuned” model differs from published results due to inaccessible weights and requires 500 Hz, 5-second *z* score normalized inputs. The training of ECG-FM was implemented with custom training code due to dependency complexities. It requires a custom library, which is only compatible with the end-of-life version of Python 3.9 (Python Software Foundation). Additionally, an ONNX export is not possible with these custom functions, impeding the usage of interoperable standards and therefore the deployment into the platform. The PyTorch implementation, as an alternative, could not be used due to the outdated and unsupported versions of major libraries, resulting in dependency conflicts. The results of ECG-FM are therefore achieved outside the platform, yet are given for comparative purposes. DSAIL SNU was adapted for ONNX export and initialized with the unavailable coinput features (age and sex) using the default values and their required missing feature flags as specified in its corresponding publication, alongside the used minimum-maximum normalization in pretraining.

All ECG recordings were processed at the sampling rate required by each model. For ECG-FM, recordings that are natively 500 Hz (PTB-XL, Yang et al , MIMIC-IV-ECG) were used unchanged, while recordings available only at 100  Hz (EDMS) were up-sampled to 500  Hz via a Fast Fourier Transform to match the model’s input requirement. DSAIL SNU, XceptionTime, and InceptionTime require a 10-second ECG sampled at 100 Hz. Consequently, any 500 Hz recordings (PTB-XL, Yang et al, MIMIC-IV-ECG) were down-sampled to 100 Hz using the platform’s interoperable data-loading pipeline, and recordings only available at 100  Hz (EDMS) were left unchanged.

### Performance Metrics

To evaluate model performance, we use the *F*_1_-score for overall assessment and calculate the average and median for central tendency across datasets. Predictions are derived by selecting the class with the highest probability. We use the *F*_1_-score rather than area under the curve because clinical relevance often requires accurate classification at a single operating threshold— outlining the critical balance between precision and recall—whereas area under the curve summarizes performance across all possible thresholds and may not reflect the real-world consequences of specific predictions. Additionally, we focus on the weighted *F*_1_-score to account for the class imbalance commonly seen in medical datasets, ensuring that minority classes are appropriately represented in the evaluation. Macro *F*_1_, accuracy, precision, recall, Brier score, and expected calibration error top-label, and classwise (macro and weighted), with bootstrapped 95% CIs, as well as per class metrics, confusion matrices, and 2-sided paired *t* tests for external datasets, are given in the Multimedia Appendix 3. For *F*_1_-score evaluation, classes not present in the PhysioNet labels were removed. To reflect realistic out-of-distribution prediction, the comparison did not remove false positives. Robustness, indicated by lower IQR and coefficient of variation (CV) values, suggests consistency across datasets. Computational scaling is analyzed using the number of parameters, floating-point operations per second (FLOPs), training, and inference timings. For all architectures on the ExChanGeAI platform, estimated timings are reported as the mean with SD, evaluated using a run of 1500 ECGs from the MIMIC database, based on the superclass subset and the respective models.

These comprehensive evaluations enable us to determine how well models within ExChanGeAI perform under varied conditions, providing insights into their practical application in diverse real-world scenarios. Through this extensive testing framework, we confirm ExChanGeAI’s robustness, adaptability, and reliability for ECG analysis across multiple datasets, diagnostic statements, and applicability for different use cases.

### Ethical Considerations

Collection and analysis of the EDMS dataset were approved by the responsible medical ethics committee (Ärztekammer Westfalen-Lippe, approval EDMS: no. 2022‐494 f-S) under a waiver of informed consent in accordance with state law for health data privacy (§6 Abs. 2 Gesundheitsdatenschutzgesetz Nordrhein Westfalen (Health Data Protection Act of North Rhine-Westphalia)). The creation and analysis of the MIMIC-IV-ECG dataset were reviewed by the Institutional Review Boards of Beth Israel Deaconess Medical Center and the Massachusetts Institute of Technology, which waived the requirement for individual patient consent because the project did not impact clinical care and all protected health information was deidentified. The Yang et al dataset was approved by the Medical Ethics Committee of Chinese People's Liberation Army General Hospital (approval no S2019-318-03) under informed consent from the participants. The PTB-XL dataset is publicly available under a waiver of the Institutional Ethics Committee (approval no PTB-2020‐1), complying with Health Insurance Portability and Accountability Act (HIPAA) standards. All waivers allow secondary analysis with given approvals under the respective data regulation and privacy protection standards.

## Results

**Figure 4.**
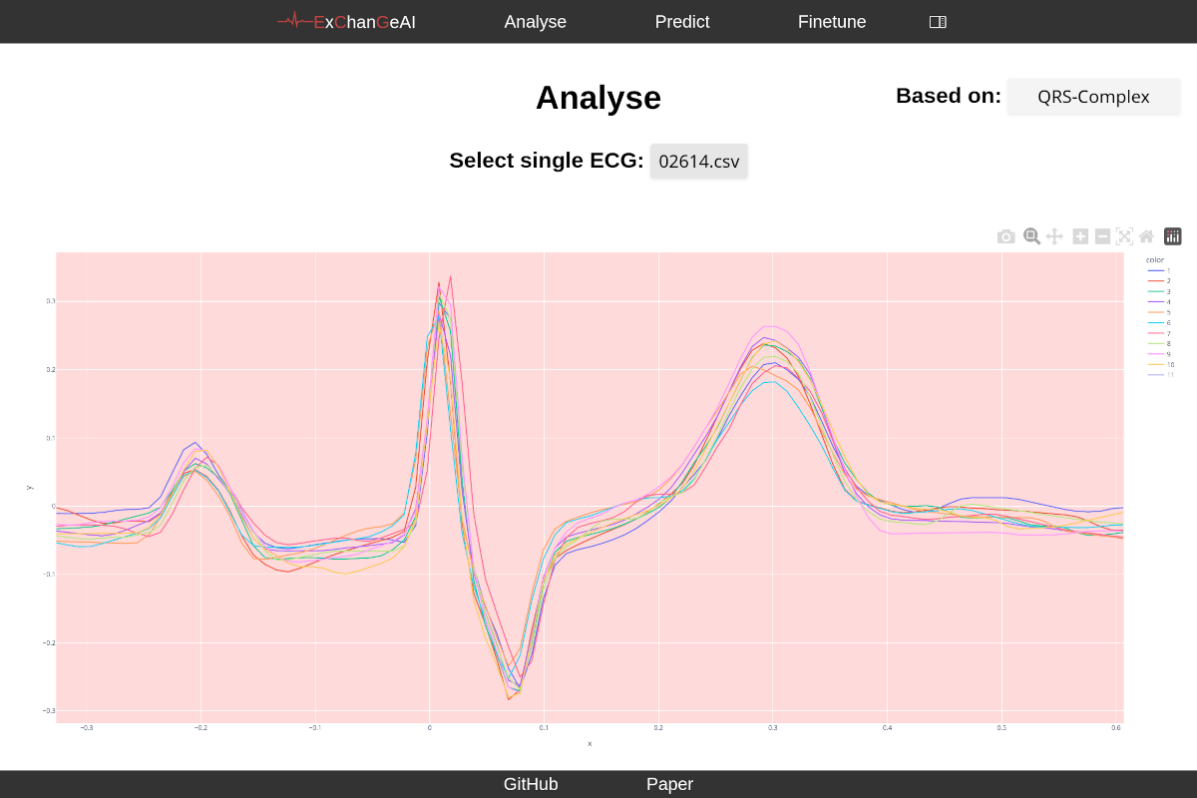
Overview of the ExChanGeAI web interface, showing the “Analyse” page with a sample electrocardiogram in QRS-waveforms. ECG: electrocardiogram.

**Figure 5.**
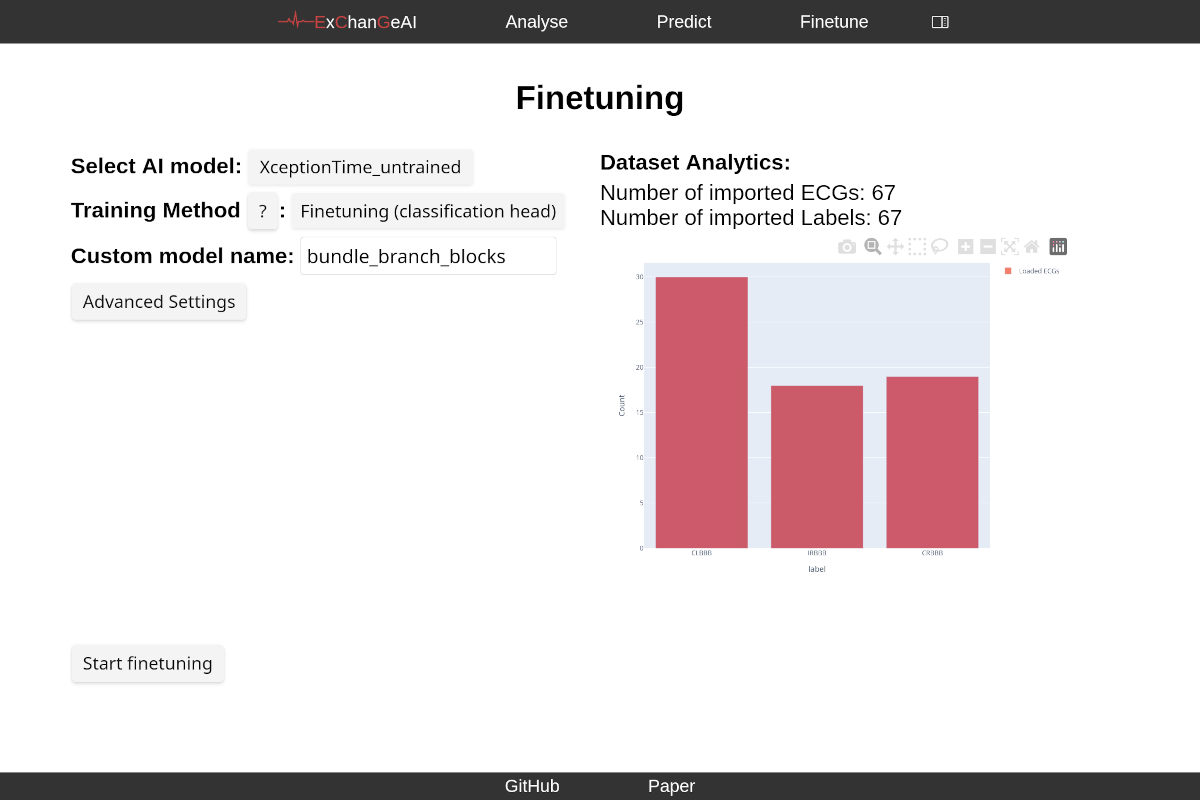
Overview of the ExChanGeAI web interface, showing the “Finetune” page, showing the default necessary parameters, and the data distribution across labels of an example bundle-branch-block dataset. ECG: electrocardiogram.

The central goal of ExChanGeAI is to make model selection and empirical comparison tractable, reproducible, and accessible within a single, seamless platform. Therefore, the interface is visually structured into different foci, such as data analysis (refer to Figure 4). The analysis view provides interactive visualization of individual ECG files. Users can select to view ECG data based on multiple views - raw time series, QRS complexes, fiducial point annotation, Rlign median beats, and Rlign time-aligned ECG. When visualizing QRS complexes, the interface displays overlaid waveforms, potentially highlighting morphological features. For raw time series visualization, the platform presents the standard 12-lead ECG signals as separate plots, allowing for detailed inspection of each lead’s waveform. The fine-tuning view displays options for model selection, training method, and custom model naming. A bar chart visualization summarizes the distribution of labels within the loaded dataset, presenting counts for categories, such as “CLBBB,” “IRBBB,” and “CRBBB” as shown in Figure 5. Numerical dataset characteristics, including the total number of imported ECGs and labels, are presented as well.

Table 3 shows the weighted *F*_1_-scores of the Xception-, InceptionTime, DSAIL SNU, and ECG-FM architectures across the different classification targets on the test datasets. The tables are organized such that columns represent different models, while rows represent various classification tasks and datasets. Specifically, each row corresponds to a particular classification task evaluated across different test sets (PTB-XL, Yang et al, MIMIC-IV, and EDMS). The most comprehensively de novo–trained model XceptionTime serves as a reference (Training PTB-XL folds 1-8) for assessing performance scaling with increased data availability.

**Table 3.** Performance evaluation of various models on electrocardiogram classification tasks across the test datasets.

Model	XceptionTime^a^^b^		InceptionTime^a^^b^	DSAIL SNU^a^^c^		ECG-FM^d^	
	Training folds (1-8)	Training folds (9)		Fine-tune	Pretrained PhysioNet 2021	Pretrained PhysioNet 2021	Fine-tune
Training PTB-XL^e^ folds	1-8	9	9	9	—^f^	—	9
Trained layers	All	All	All	Head	—	—	Head
Superclasses							
PTB-XL	0.792	0.686^g^	0.651	0.536^h^	0.038^h^	0.174^h^	0.690^i^^,^^h^
Yang et al	0.335	0.647^i^	0.064	0.585^g^	0.402	0.491	0.173
MIMIC-IV^j^	0.371	0.374^i^	0.358^g^	0.323	0.055	0.192	0.355
EDMS^k^	0.432	0.387^i^	0.379^g^	0.331	0.039	0.333	0.216
Myocardial infarcts							
PTB-XL	0.938	0.853^g^	0.902^i^	0.685^h^	—	—	0.685^h^
MIMIC-IV	0.753	0.734^i^	0.726^g^	0.566	—	—	0.403
EDMS	0.566	0.484	0.584^i^	0.343	—	—	0.532^g^
Bundle branch blocks							
PTB-XL	0.911	0.912^i^	0.891^g^	0.832^h^	0.000^h^	0.016^h^	0.790^h^
Yang et al	0.730	0.101	0.792^i^	0.496	0.007	0.089	0.617^g^
MIMIC-IV	0.825	0.820^i^	0.819^g^	0.333	0.000	0.028	0.087
EDMS	0.739	0.827^i^	0.732^g^	0.622	0.000	0.118	0.248
Revascularization							
EDMS	0.750^l^	0.688^i^	0.645^g^	0.635	—	—	0.603
Weighted *F*_1_ (interdataset only; excluding PTB-XL)							
Mean (SD)↑	0.611 (0.188)	0.562^g^ (0.243)	0.567^i^ (0.251)	0.470 (0.137)	—	—	0.359 (0.193)
Median (IQR)↑	0.73 (0.432-0.750)	0.647^i^ (0.387-0.734)	0.645^g^ (0.379-0.732)	0.496 (0.333-0.585)	—	—	0.355 (0.216-0.532)
CV^m^↓	0.308	0.433^g^	0.443	0.290^i^	—	—	0.538

a Trained via ExChanGeAI.

b De novo training.

c DSAIL SNU: Data Science & Artificial Intelligence Laboratory Seoul National University

d ECG-FM: Electrocardiogram Foundation Model; used custom code for the training platform.

e PTB-XL: Physikalisch-Technischen Bundesanstalt-extra large.

f Not applicable.

g Second best model in this category.

h These models were pre-trained on datasets including PTB-XL; results on this specific target should be interpreted as in-distribution evaluations.

i Best model in this category.

j MIMIC-IV: Medical Information Mart for Intensive Care IV.

k EDMS: Emergency Department Münster.

l Transfer learning based on reference XceptionTime (superclasses with folds 1‐8).

m CV: coefficient of variation.

For illustration, the first row presents superclass classification scores on the PTB-XL test dataset, whereas the reference XceptionTime (trained on PTB-XL folds 1‐8) achieves a weighted *F*_1_-score of 0.792, and XceptionTime, trained on fold 9 only, reaches 0.686. As an example for MI, the InceptionTime model achieves the second-best *F*_1_-score across both inter-datasets (0.726 on MIMIC-IV and 0.584 on EDMS), while XceptionTime achieves a slightly higher score on the former (0.734), and ECG-FM on the latter (0.532). The last rows show the aggregated statistics, showing Xception and InceptionTime have the best average and median *F*_1_-scores, outlining the top overall performing models, while DSAIL SNU shows the best IQR and CV values, exhibiting the most robust scores across external datasets. As anticipated, increasing the amount of training data leads to improved performance. Importantly, ExChanGeAI is able to handle this scalability, achieving better outcomes as more data are incorporated (see reference model “XceptionTime” in Table 3). For example, on the PTB-XL dataset, test performance on the superclasses and myocardial infarct targets improves substantially—by 15.4% and 9.96% respectively—when XceptionTime is trained on folds 1‐8 compared to training only on fold 9.

XceptionTime and InceptionTime, representing architectures trained de novo on PTB-XL*,* meaning with random initialization and without any prior training at all, often achieved the highest results across classification tasks. In contrast, the pretrained models, DSAIL SNU and ECG-FM, exhibited a more nuanced performance profile in our limited data setting. Initially, both models demonstrated suboptimal classification accuracy, especially on datasets outside of their pretraining domain (PhysioNet 2021). Fine-tuning them on a single PTB-XL fold for each classification target led to significant improvements for both DSAIL SNU and ECG-FM. However, they were outperformed by the de novo InceptionTime (8 out of 9 targets) and XceptionTime models (7 out of 9) within our evaluation. Still, fine-tuned DSAIL SNU exhibited the best robustness (lowest IQR and CV), suggesting stable results across disparate external cohorts despite lower mean and median *F*_1_-scores. This increase in intradataset performance does not always translate to interdataset performance, as expected, due to overfitting to the dataset distribution. Overall, XceptionTime and InceptionTime trained from scratch showed the highest average and median *F*_1_-scores across all evaluated classification tasks.

The InceptionTime model trained on folds 1‐8, as reported in the PTB-XL benchmark paper [46], achieves a macro *F*_1_-score of 0.7495. In comparison, our reference XceptionTime model attains a macro *F*_1_-score of 0.768, while a comparable training (not in Table 3) of InceptionTime achieves 0.7707. These results demonstrate that ExChanGeAI’s training capabilities not only match but also surpass established benchmark baselines, all without reliance on additional resources. Additionally, the platform’s flexible workflow enables rapid prototyping for novel tasks, such as revascularization prediction (absent in PTB-XL), trained and evaluated using the new EDMS dataset. Here, transfer learning, based upon the reference XceptionTime (0.750), increased the *F*_1_-score by 9% relative to de novo XceptionTime (0.688).

Additionally, it has to be noted that the foundation model ECG-FM is the largest with over 90 million parameters, followed by DSAIL SNU (2M), Xception (401K), and InceptionTime (457K). In terms of computational complexity, ECG-FM is the most demanding (14 GFLOPS), followed in descending order by Inception-(460 MFLOPS), XceptionTime (256 MFLOPS), and DSAIL SNU (89 MFLOPS). The inference timings on a 6-core Zen4 CPU correspond to mean 27 (SD 33.78) ms (XceptionTime), mean 26 (SD 36.56) ms (InceptionTime), and mean 29.5 (SD 13.9) ms (DSAIL) using the ExChanGeAI platform. Training with 1500 training samples is estimated with mean 13180 (SD 44) ms per epoch and 8.79 ms per sample (XceptionTime), mean 19520 (SD 153) ms per epoch and 13.01 ms per sample (InceptionTime), and mean 10210 (SD 34) ms per epoch and 6.80 ms per sample (DSAIL). Comparing the classification performance against the computational complexity, XceptionTime and InceptionTime stand out as the top performers. All models trained on the PTB-XL dataset are available in Multimedia Appendix 4.

## Discussion

### Overview

Our evaluation of ExChanGeAI on established architectures reveals several key insights into model selection, particularly in data-constrained scenarios. The end-to-end platform streamlines both training and fine-tuning, yielding robust performance metrics across diverse ECG classification tasks.

### Comparison With Prior Work

Our evaluation shows training on limited data and cross-dataset testing exposes inherent generalization gaps and variability in performance—a major difference compared to the often overoptimistic intradataset results seen in the literature. Consequently, the near-perfect accuracy metrics—in intratest set and simple tasks, such as tachy- and bradycardia prediction [28]—are not reproducible when models are evaluated on external, independent datasets. However, when models are evaluated using intradataset testing and ample training data are available, achieving high scores becomes more feasible and reproducible on external datasets (see the baseline XceptionTime model in the Multimedia Appendix 3). As expected and in line with previous findings [52,53], all models exhibited performance drops on external datasets. Yet, as an important factor, the end-to-end platform training surpasses the established benchmark InceptionTime model, outlining the competitive training performance of the platform without requiring expert knowledge.

### Principal Findings

XceptionTime models were particularly notable for their parameter efficiency and competitive accuracy, reaffirming their architectural strength. Notably, learning from scratch proved to be a strong alternative to transfer learning, as de novo XceptionTime and InceptionTime models often outperformed fine-tuned pretrained models despite having fewer parameters. However, performance variability was observed across different classification tasks and datasets, as expected, indicating a sensitivity to dataset-specific scaling and parameter optimization within specific model architectures. Pretrained models, while anticipated to leverage their extensive prior knowledge, presented a mixed picture in our data-limited scenarios: while fine-tuning improved their performance, they generally did not consistently surpass the de novo trained XceptionTime and InceptionTime models. However, the pretrained model did exhibit enhanced robustness against performance degradation across external datasets in most cases, compared to de novo trained models. Among all, DSAIL SNU demonstrated the lowest performance variance, underscoring its robustness.

### Limitations

First, while pretrained models offer potential advantages, their benefits are not guaranteed in data-constrained scenarios. Training from scratch within ExChanGeAI frequently yielded top results. This underscores the critical importance of empirical validation and careful model selection tailored to each dataset and use case. Second, the inherent influence of model architecture on performance, coupled with the relative consistency of the subsequent training process across architectures, underscores the value of an end-to-end platform that simplifies exploration and deployment of diverse, yet effective, models. Third, while the evaluation was conducted using data-constrained scenarios, rigorous validation across diverse external datasets and the baseline comparison model also outlines the advantage of more training samples; however, these may be difficult to obtain in a clinical setting. Fourth, while the given models can be trained outside the platform, with even more customization, the usage of ExChanGeAI reduces many technical burdens, facilitating faster deployment as it eliminates the need for code for data ingestion, preprocessing, training, and evaluation for new models. Fifth, all evaluations have been conducted with a 100 Hz sampling rate by default, which, according to multiple research papers, does not notably decrease the classification performance. However, downsampling may lead to a loss of high-frequency clinical details, such as fragmentation and notches. Researchers should be aware that this loss of fidelity may be critical for specific pathologies not covered in the current classification tasks, though the platform allows for higher sampling rates if required. Sixth, defining “revascularization” by treatment status serves as a proxy for actionable clinical need. While this implies that the label incorporates medical decision-making alongside pathology, predicting this outcome remains a clinically vital advancement for identifying patients requiring urgent intervention. Seventh, while the platform supports seamless deployment of ONNX-compatible architectures, we acknowledge that integrating foundation models with external dependencies or specific libraries (eg, ECG-FM) currently requires execution via external scripts rather than the native end-to-end platform. Eighth, the DSAIL SNU model replaces missing age/sex with default values and missing flag indicators, exactly as it was pretrained. If demographic data were available, its performance may improve beyond the results reported here. Finally, we contributed to the evaluation of the novel revascularization task using a stratified 20% hold-out subset of our EDMS cohort. Consequently, these new results can serve as an internal validation only, and the generalizability to external cohorts cannot be guaranteed.

### Future Work

While acknowledging potential limitations for expert users seeking highly specialized customizations, the platform’s modular design allows for the future integration of additional compatible architectures, expanding its versatility. The main focus of possible future work could be the integration of explainable or interpretable ML, including its visualization for each prediction.

### Conclusions

A major strength of ExChanGeAI is its ability to democratize advanced deep learning for ECG analysis. By integrating pretrained, fine-tuned, and untrained models within a unified interface, ExChanGeAI overcomes significant barriers associated with data loading, model-specific installation, environment setup, and code dependencies, particularly benefiting nonexperts and general-purpose applications. This not only enables rapid prototyping and empirical validation by both experts and nonexperts but also encourages open science and sharing of ready-to-use models for collaborative research. Ultimately, ExChanGeAI aims to enhance the accessibility of deep learning models and reduce operational overhead, facilitating broader adoption and accelerating progress. This approach not only minimizes human error and technical debt but also supports best practices for reproducible research and clinical validation. Limitations are mainly posed by the available data and infrastructure, even though the training, on modern machines, becomes significantly easier due to the large increase in computational power in recent years and wider adoption of specialized hardware, such as GPUs and neural processing units.

In conclusion, this work introduced ExChanGeAI, a novel open-source platform designed to streamline and democratize the application of deep learning for ECG analysis. Our results demonstrate the effectiveness of ExChanGeAI across both conventional and state-of-the-art deep learning models—even with limited data—and highlight that pretrained models are not always superior in data-constrained scenarios. Regular empirical benchmarking and model selection remain crucial. By promoting accessibility, reproducibility, and systematic model comparison, ExChanGeAI broadens participation in deep learning research and clinical adoption in ECG analysis.

## Supplementary material

10.2196/81116 Multimedia Appendix 1 Sample size and class distribution for training samples of PTB-XL (Physikalisch-Technischen Bundesanstalt-extra large [National Metrology Institute of Germany]) across defined classification targets.

10.2196/81116 Multimedia Appendix 2 Overview of dataset characteristics, including the total number of ECG recordings, age, and sex distribution for the PTB-XL (Physikalisch-Technischen Bundesanstalt-extra large [National Metrology Institute of Germany]), MIMIC-IV-ECG (Medical Information Mart for Intensive Care IV Electrocardiogram), Yang et al., and EDMS (Emergency Department Münster) datasets.

10.2196/81116 Multimedia Appendix 3 Extended classification and calibration metrics of the trained models on electrocardiogram classification tasks across the external datasets.

10.2196/81116 Multimedia Appendix 4 Trained and fine-tuned models using the ExChanGeAI platform based on the PTB-XL (Physikalisch-Technischen Bundesanstalt-extra large [National Metrology Institute of Germany]) dataset. Training conducted using default settings (commit number 4d862c04) to ensure reproducibility.

## Abbreviations

AI: artificial intelligence
BBB: bundle branch blocks
CLBBB: complete left bundle branch block
CNN: convolutional neural network
CRBBB: complete right bundle branch block
CV: coefficient of variation
DICOM: Digital Imaging and Communication in Medicine
DSAIL SNU: Data Science & Artificial Intelligence Laboratory Seoul National University
ECG: electrocardiogram
ECG-FM: Electrocardiogram Foundation Model
EDMS: Emergency Department Münster
FLOP: floating-point operations per second
GAMA: General Automated Machine Learning Assistant
GPU: graphics processing unit
HIPAA: Health Insurance Portability and Accountability Act
HYP: hypertrophy
*ICD-10*: *International Classification of Diseases, 10th Revision*
ILBBB: incomplete left bundle branch block
MI: myocardial infarction
MIMIC-IV-ECG: Medical Information Mart for Intensive Care IV Electrocardiogram
MIT: Massachusetts Institute of Technology
ONNX: Open Neural Network Exchange
PT-XL: Physikalisch-Technischen Bundesanstalt-extra large
STTC: ST/T changes

## References

[1] Lin CS, Liu WT, Tsai DJ (2024). AI-enabled electrocardiography alert intervention and all-cause mortality: a pragmatic randomized clinical trial. Nat Med.

[2] Adedinsewo DA, Morales-Lara AC, Afolabi BB (2024). Artificial intelligence guided screening for cardiomyopathies in an obstetric population: a pragmatic randomized clinical trial. Nat Med.

[3] Sau A, Pastika L, Sieliwonczyk E (2024). Artificial intelligence-enabled electrocardiogram for mortality and cardiovascular risk estimation: a model development and validation study. Lancet Digit Health.

[4] Widder DG, Whittaker M, West SM (2024). Why “open” AI systems are actually closed, and why this matters. Nature New Biol.

[5] Polevikov S (2023). Advancing AI in healthcare: a comprehensive review of best practices. Clin Chim Acta.

[6] Rahman M, Victoros E, Ernest J, Davis R, Shanjana Y, Islam M (2024). Impact of artificial intelligence (AI) technology in the healthcare sector: a critical evaluation of both sides of the coin. Clin Med Insights Pathol.

[7] Rauniyar A, Hagos DH, Jha D (2024). Federated learning for medical applications: a taxonomy, current trends, challenges, and future research directions. IEEE Internet Things J.

[8] Jang JH, Kim TY, Yoon D (2021). Effectiveness of transfer learning for deep learning-based electrocardiogram analysis. Healthc Inform Res.

[9] Weimann K, Conrad TOF (2021). Transfer learning for ECG classification. Sci Rep.

[10] Chato L, Regentova E (2023). Survey of transfer learning approaches in the machine learning of digital health sensing data. J Pers Med.

[11] Avetisyan A, Tigranyan S, Asatryan A (2024). Deep neural networks generalization and fine-tuning for 12-lead ECG classification. Biomed Signal Process Control.

[12] Kaplan Berkaya S, Uysal AK, Sora Gunal E, Ergin S, Gunal S, Gulmezoglu MB (2018). A survey on ECG analysis. Biomed Signal Process Control.

[13] Ebrahimi Z, Loni M, Daneshtalab M, Gharehbaghi A (2020). A review on deep learning methods for ECG arrhythmia classification. Expert Syst Appl: X.

[14] Qin K, Huang W, Zhang T, Zhang H, Cheng X (2024). A lightweight SelfONN model for general ECG classification with pretraining. Biomed Signal Process Control.

[15] Zhang S, Fang Y, Ren Y (2024). ECG autoencoder based on low-rank attention. Sci Rep.

[16] Roy M, Majumder S, Halder A, Biswas U (2023). ECG-NET: a deep LSTM autoencoder for detecting anomalous ECG. Eng Appl Artif Intell.

[17] Thambawita V, Isaksen JL, Hicks SA (2021). DeepFake electrocardiograms using generative adversarial networks are the beginning of the end for privacy issues in medicine. Sci Rep.

[18] Singh P, Sharma A (2022). Attention-based convolutional denoising autoencoder for two-lead ECG denoising and arrhythmia classification. IEEE Trans Instrum Meas.

[19] Goldberger AL, Amaral LAN, Glass L (2000). PhysioBank, PhysioToolkit, and PhysioNet. Circulation.

[20] Wagner P, Strodthoff N, Bousseljot RD (2020). PTB-XL, a large publicly available electrocardiography dataset. Sci Data.

[21] Bickmann L, Plagwitz L, Varghese J (2023). Post Hoc sample size estimation for deep learning architectures for ECG-classification. Stud Health Technol Inform.

[22] Ismail Fawaz H, Lucas B, Forestier G (2020). InceptionTime: finding AlexNet for time series classification. Data Min Knowl Disc.

[23] Rahimian E, Zabihi S, Atashzar SF, Asif A, Mohammadi A XceptionTime: independent time-window xceptiontime architecture for hand gesture classification.

[24] Han Y, Murino V, Liu X, Zhang X, Ding C (2025). A systematic review on foundation models for electrocardiogram analysis: initial strides and expansive horizons. arXiv.

[25] Li J, Aguirre A, Moura J (2025). An electrocardiogram foundation model built on over 10 million recordings with external evaluation across multiple domains. arXiv.

[26] Wang Y, Cao X, Hu Y (2025). AnyECG: foundational models for multitask cardiac analysis in real-world settings. arXiv.

[27] Zhang S, Du Y, Wang W (2025). ECGFM: a foundation model for ECG analysis trained on a multi-center million-ECG dataset. Inf Fusion.

[28] McKeen K, Masood S, Toma A, Rubin B, Wang B (2025). ECG-FM: an open electrocardiogram foundation model. JAMIA Open.

[29] Mathew G, Barbosa D, Prince J, Venkatraman S (2024). Foundation models for cardiovascular disease detection via biosignals from digital stethoscopes. npj Cardiovasc Health.

[30] Demšar J, Curk T, Erjavec A (2013). Orange: data mining toolbox in python. J Mach Learn Res.

[31] Gijsbers P, Vanschoren J (2019). GAMA: Genetic automated machine learning assistant. J Open Source Softw.

[32] Bickmann L ExChanGeAI. GitHub.

[33] Makowski D, Pham T, Lau ZJ (2021). NeuroKit2: a Python toolbox for neurophysiological signal processing. Behav Res Methods.

[34] Plagwitz L, Bickmann L, Fujarski M (2024). The Rlign algorithm for enhanced electrocardiogram analysis through R-peak alignment for explainable classification and clustering. arXiv.

[35] Salimi A, Kalmady SV, Hindle A, Zaiane O, Kaul P (2025). Exploring best practices for ECG signal processing in machine learning. arXiv.

[36] Mehari T, Strodthoff N (2023). Towards quantitative precision for ECG analysis: leveraging state space models, self-supervision and patient metadata. IEEE J Biomed Health Inform.

[37] Lee KS, Park HJ, Kim JE (2022). Compressed deep learning to classify arrhythmia in an embedded wearable device. Sensors (Basel).

[38] Büscher A, Plagwitz L, Yildirim K (2025). Deep learning electrocardiogram model for risk stratification of coronary revascularization need in the emergency department. Eur Heart J.

[39] Reyna MA, Sadr N, Alday EAP Will two do? varying dimensions in electrocardiography: the physionet/computing in cardiology challenge 2021.

[40] Han H, Park S, Min S Towards high generalization performance on electrocardiogram classification.

[41] Ansel J, Yang E, He H (2024). PyTorch 2: faster machine learning through dynamic python bytecode transformation and graph compilation.

[42] Loshchilov I, Hutter F (2019). Decoupled Weight Decay Regularization. https://openreview.net/forum?id=Bkg6RiCqY7.

[43] Zhou P, Xie X, Lin Z, Yan S (2024). Towards understanding convergence and generalization of AdamW. IEEE Trans Pattern Anal Mach Intell.

[44] Kingma DP, Ba J (2017). Adam: a method for stochastic optimization. arXiv.

[45] Li J, Yang X A cyclical learning rate method in deep learning training.

[46] Strodthoff N, Wagner P, Schaeffter T, Samek W (2021). Deep learning for ECG analysis: benchmarks and insights from PTB-XL. IEEE J Biomed Health Inform.

[47] Lai J, Tan H, Wang J (2023). Practical intelligent diagnostic algorithm for wearable 12-lead ECG via self-supervised learning on large-scale dataset. Nat Commun.

[48] Yang W, Feng Q (2023). Offline test set of ECG multi-label classfication.

[49] Gow B, Pollard T, Nathanson LA MIMIC-IV-ECG: diagnostic electrocardiogram matched subset.

[50] Frénay B, Verleysen M (2014). Classification in the presence of label noise: a survey. IEEE Trans Neural Netw Learn Syst.

[51] Reyna MA, Sadr N, Perez Alday EA (2022). Issues in the automated classification of multilead ecgs using heterogeneous labels and populations. Physiol Meas.

[52] Martínez-Sellés M, Marina-Breysse M (2023). Current and future use of artificial intelligence in electrocardiography. J Cardiovasc Dev Dis.

[53] McDermott MBA, Wang S, Marinsek N, Ranganath R, Foschini L, Ghassemi M (2021). Reproducibility in machine learning for health research: still a ways to go. Sci Transl Med.

