# Supplementary material for: End-to-End Platform for Electrocardiogram Analysis and Model Fine-Tuning: Development and Validation Study
Source: J Med Internet Res. 2026 Jan 30;28:e81116. doi: 10.2196/81116 (PMC12858047; doi:10.2196/81116)
Supplement: Multimedia Appendix 1 [file jmir-v28-e81116-s001.pdf]

Table S1: Sample size and class distribution for training samples of PTB-XL across defined classification targets. Fold 1-8 is used for baseline models, fold 9 for training or fine-tuning, and fold 10 for testing.

	Superclasses				Myocardial infarcts		Bundle branch blocks		
Stratified fold	MI	CD	STTC	HYP	AMI	IMI	CLBBB	CRBBB	IRBBB
1-8	2.043	1.353	1.905	415	576	984	285	75	257
Total	5.716				1.560		617		
9	233	171	254	64	58	123	30	19	18
Total	722				181		67		
10	256	184	242	56	66	139	38	12	36
Total	738				205		86		

This is a Multimedia Appendix to a full manuscript published in the J Med Internet Res. For full copyright and citation information see http://dx.doi.org/10.2196/jmir.81116.
