# Supplementary material for: End-to-End Platform for Electrocardiogram Analysis and Model Fine-Tuning: Development and Validation Study
Source: J Med Internet Res. 2026 Jan 30;28:e81116. doi: 10.2196/81116 (PMC12858047; doi:10.2196/81116)
Supplement: Multimedia Appendix 2 [file jmir-v28-e81116-s002.pdf]

Table S2: Overview of dataset characteristics, including the total number of ECG recordings, age distribution (median and interquartile range (IQR) or mean ± standard deviation (SD) where available), and sex distribution (percentage of male, female, and unknown/diverse subjects) for the PTB-XL, MIMIC-IV ECG, Yang et al., and EDMS datasets. This summary highlights differences in cohort size and demographic composition among the datasets.

Dataset	#ECGs	Age		Sex distribution (%)		
		Median (IQR)	Mean ± SD	Male	Female	Unknown/Diverse
MIMIC-IV ECG	800.035	66 (23)	-	50.77	48.67	0.56
PTB-XL	21.799	62 (22)	63 ± 32	52	48	-
EDMS	18.673	-	55 ± 21	50.84	49.15	0.02
Yang et al.	7.000	-	51	67.3	32.8	-

This is a Multimedia Appendix to a full manuscript published in the J Med Internet Res. For full copyright and citation information see http://dx.doi.org/10.2196/jmir.81116.
